# Factors associated with pain after non-surgical treatment for trapeziometacarpal joint osteoarthritis

**DOI:** 10.1177/17531934261459874

**Published:** 2026-06-25

**Authors:** Lisa M J Esteban Lopez, Lisa Hoogendam, Ruud W Selles, the THETA Study Group Collaborators, Raymond van Adrichem, Robbert M Wouters

**Affiliations:** 1 Department of Plastic, Reconstructive, and Hand Surgery, Erasmus MC, University Medical Center Rotterdam, The Netherlands; 2 Department of Rehabilitation Medicine, Erasmus MC, University Medical Center Rotterdam, The Netherlands; 3 Center for Hand Therapy, Xpert Handtherapie, Eindhoven, The Netherlands; 4 Xpert Clinics Hand and Wrist Care, Eindhoven, The Netherlands

**Keywords:** Association, osteoarthritis, pain, therapy, thumb

## Abstract

**Introduction:**

Although non-surgical treatment for trapeziometacarpal joint (TMJ) osteoarthritis (OA) reduces pain, outcomes vary substantially. This study investigated factors associated with pain at 3 months after starting non-surgical treatment for TMJ OA.

**Methods:**

This secondary analysis from a previous randomized controlled trial included 113 patients (Eaton–Glickel stage ⩾2 TMJ OA) from 18 outpatient hand surgery clinics in The Netherlands, comprising specialized clinics, regional hospitals and one academic hospital. Participants were randomized to orthosis-only or orthosis + exercise groups. Both groups received a standardized custom-made thumb orthosis, and the combined group also received supervised hand therapy with a structured home exercise programme. The primary outcome was the Michigan Hand outcomes Questionnaire (MHQ) pain subscale at 3 months, which was modified so that higher scores indicated less pain. We used linear regression analysis to investigate the association of baseline pain, psychological factors, outcome expectations, adherence to therapy, perceived attention, patient experience with care, treatment frequency and changes in grip and pinch strength with MHQ pain.

**Results:**

Better MHQ pain score at baseline, higher baseline outcome expectations and improvement in grip strength after 3 months were associated with better pain outcomes. The MHQ pain baseline score had the strongest independent association.

**Conclusion:**

Baseline pain severity, outcome expectations and grip strength are associated with pain at 3 months after the use of orthoses with or without exercise therapy for TMJ OA.

**Level of evidence:**

II

## Introduction

Trapeziometacarpal joint (TMJ) osteoarthritis (OA) is a common hand condition with a symptomatic prevalence of 7% in women and 2% in men over the age of 50 (Haugen et al., 2011). Radiographic TMJ OA is even more prevalent, indicating that many individuals have TMJ OA without seeking medical care (van der Oest et al., 2021). Symptomatic disease may result in pain, limitations in activities of daily living and reduced quality of life (Haugen et al., 2011). Given the functional and personal burden associated with TMJ OA and the expected rise in prevalence owing to an ageing population, identifying effective treatment strategies is increasingly important.

Current guidelines for treating TMJ OA recommend non-surgical interventions, such as analgesics, intra-articular injections, orthoses, joint protection programmes and exercise therapy, before considering surgery (Bertozzi et al., 2015; Buhler et al., 2019; Estee et al., 2024; Gangopadhyay et al., 2025; Kolasinski et al., 2020; Kroon et al., 2016; Sankah et al., 2019; Spaans et al., 2015).

Although the use of orthoses, exercise therapy and a combination of both have demonstrated efficacy in reducing pain, outcomes such as pain and function vary substantially between studies and patient groups (Esteban Lopez et al., 2023; Karanasios et al., 2024; Tsehaie et al., 2018; Wouters et al., 2019). This raises the question about which factors influence pain reduction beyond the treatment itself. Most previous research on variance in outcomes reported relatively low variance for patient characteristics, baseline pain and psychological factors such as pain catastrophizing and psychological distress (Tsehaie et al., 2018; van der Oest et al., 2022; Wouters et al., 2019). This suggests that key influencing factors may not have been adequately considered or measured. Hypothetically, factors such as adherence to therapy, perceived attention during treatment and frequency of therapy sessions may also contribute to treatment outcomes. These are rarely studied in TMJ OA, leaving a critical gap in understanding the mechanisms underlying pain reduction. Moreover, these aspects could be influenced and optimized by clinicians, potentially improving outcomes. A better understanding of these factors may also contribute to decision-making about treatment, by identifying patients who are likely to benefit from continued non-surgical management and those in whom surgical intervention may ultimately be considered.

We have investigated the association of baseline pain, psychological factors, outcome expectations, adherence to therapy, perceived attention, patient experience with care, treatment frequency and change in grip and pinch strength with pain at 3 months after starting non-surgical treatment.

## Methods

### Study design

As this was a secondary analysis of a multicentre randomized controlled trial (RCT) and was focused on associations between various factors and pain at 3 months, rather than comparing differences in treatment outcomes we followed the Strengthening the Reporting of Observational Studies in Epidemiology reporting guideline (von Elm et al., 2014). All patients provided informed consent and the institutional review board approved the study (reference no. MEC-2019-0616).

### Setting

Data were collected as part of an RCT investigating the effectiveness and costs of non-surgical treatment of TMJ OA (ClinicalTrials.gov ID no. NCT05772715) between September 2020 and December 2023 from 18 outpatient hand surgery clinics in the Netherlands (Wouters et al., 2026). These included specialized clinics, regional hospitals and an academic hospital. Data were collected using Gemstracker (Erasmus MC Equipe Zorgbedrijven, 2023).

### Participants

All patients were diagnosed with TMJ OA by a hand surgeon and referred to a hand therapist for trial participation. Patients were eligible if they were adults with Eaton–Glickel stage ⩾2 TMJ OA (Eaton et al., 1985). Exclusion criteria were secondary TMJ OA, comorbidity that interfered with treatment or outcome, surgery in the medical history that interfered with treatment or outcome, previous treatment for TMJ OA, steroid injection in the hand or wrist <6 weeks before admission, and inability to understand written and spoken Dutch or English language. After an eligibility check and a >24 h consideration period, patients were included and randomized (1:1) to orthosis-only or orthosis + exercise groups.

An additional criterion for the current study was the availability of complete data for all 13 investigated factors and the primary outcome.

### Measurements

Descriptive variables recorded at baseline included age, sex, hand affected, duration of symptoms, Eaton–Glickel stage, dominant hand, type of work and educational level.

The primary outcome was the Michigan Hand outcomes Questionnaire (MHQ) pain subscale at 3 months. Scores range from 0 to 100 (we converted the pain subscale so that higher scores indicate better outcomes). The MHQ has high internal validity, consistency and acceptable reliability (Poole, 2011).

A total of 13 factors were investigated for their potential association with the MHQ pain subscale score at 3 months. Rather than focusing solely on baseline predictors, we aimed to take a broader approach by investigating variables measured at different timepoints.

Baseline pain was assessed using the MHQ pain subscale. Psychological factors were measured at baseline using the Generalized Anxiety Disorder 7-item (GAD-7), the Brief Illness Perception Questionnaire (B-IPQ), the Pain Catastrophizing Scale (PCS) and the Patient Health Questionnaire-9 (PHQ-9). These questionnaires are validated for measuring generalized anxiety, illness perception, pain catastrophizing and severity of depression, respectively (Kroenke et al., 2001; Leysen et al., 2015; Spitzer et al., 2006; Sullivan et al., 1995).

Outcome expectations were assessed at 3 months using the Credibility and Expectancy Questionnaire (CEQ) at baseline (Mertens et al., 2017; Takasaki et al., 2024). The CEQ consists of six questions, with treatment expectancy assessed by items 4–6. For the current analysis, only the scores of the outcome expectations were included, as this reflects anticipated improvement before treatment and was considered most relevant to our research question. The expectancy subscale score ranges from 3 to 27, with higher scores indicating higher expectancy.

Adherence to therapy was assessed using the patients’ self-reported adherence, measured with the Therapy Adherence Assessment Tool (TAAT), at 6 weeks (Skirven et al., 2020). This scores adherence to exercise instructions, modifying activities as instructed, using orthoses as instructed, putting full effort into the therapy and completing therapy at home on a five-point scale. Perceived attention, reflecting the patients’ perception of how attentively and empathetically the therapist listened and responded during consultations, was assessed using the Consultation and Relational Empathy Measure (CARE) at 6 weeks, with 10 items rated on a five-point scale (Mercer et al., 2004; van Dijk et al., 2017). Higher outcomes indicate greater perceived relational empathy.

Patient experience with care was measured using the Dutch validated Patient-Reported Experience Measure physical therapy questionnaire at 3 months (Heeren et al., 2016). Patients answered the question: ‘What score would you give to the care provided by your physical therapist?’ giving scores from 0 to 10, with 10 indicating excellent care.

Treatment frequency was defined as the total number of hand therapy visits during the 3 month treatment period. Changes in grip and pinch strength were measured using the methods described by Mathiowetz et al. (Allen and Barnett, 2011; Mathiowetz et al., 1984, 1985) and calculated by subtracting baseline measurements from those at 3 months.

### Intervention

Participants in the trial were randomly assigned to the orthosis-only or the orthosis + exercise group. To standardize treatment in both groups, trained hand therapists followed detailed treatment protocols (Appendix I, available online). Both groups received a custom-made thermoplastic orthosis that immobilized the TMJ in extension–abduction and the thumb metacarpophalangeal joint in slight flexion and were instructed to use the orthosis 24/7 in the first 2 weeks. Usage during light activities was reduced from 2 weeks onwards. From 6 weeks onwards, the orthosis was used only during heavy activities if necessary.

The orthosis + exercise group also had up to 12 weekly 25–30-minute physical therapy sessions, including education and daily exercises, to achieve a more stable thumb position. Exercises targeted the following muscles: abductor pollicis brevis and longus, opponens pollicis, extensor pollicis brevis and flexor pollicis brevis. For the first 6 weeks, the focus was on coordinative and selective muscle activation. In the subsequent 6 weeks, the focus was on improving thenar strength and enhancing active stability with greater force.

### Sample size and statistical methods

As the initial RCT (Wouters et al., 2026) showed no difference in residual pain between the two treatment groups we used multiple linear regression analyses on complete cases for the combined groups to investigate the independent associations of the described factors with the MHQ pain subscale at 3 months. A *p*-value of ⩽0.05 was considered to be statistically significant. Assumptions of linear regression were checked, including linearity, homoscedasticity, normality, independence of residuals and multicollinearity. No deviations were found. We assessed the standardized beta coefficient to determine which variable had the strongest association.

A non-responder analysis was done to investigate whether patients with missing values differed from those without missing values by comparing the demographics and the primary outcome between patients with data on the studied variables (responders) and those without these data (non-responders) (de Groot et al., 2011). We also performed a Little’s test (Little, 1988) to assess the null hypothesis that the data were missing completely at random.

A *post-hoc* power analysis for a multiple linear regression with 113 participants and 13 predictors found an effect size (Cohen’s *f*²) of 1.24, indicating a large effect size (Cohen, 1988; Faul et al., 2009). With an *α* of 0.05, the power was >0.99.

R (version 4.2.2; R Foundation for Statistical Computing) was used for all analyses.

## Results

### Participants

We included 166 participants in the original trial. Of these, complete data for the present analysis were available for 113 participants (60 in the orthosis-only group and 53 in the orthosis + exercise group) (Figure 1). Demographic characteristics of the study participants are presented in Table 1.

**Figure 1.**
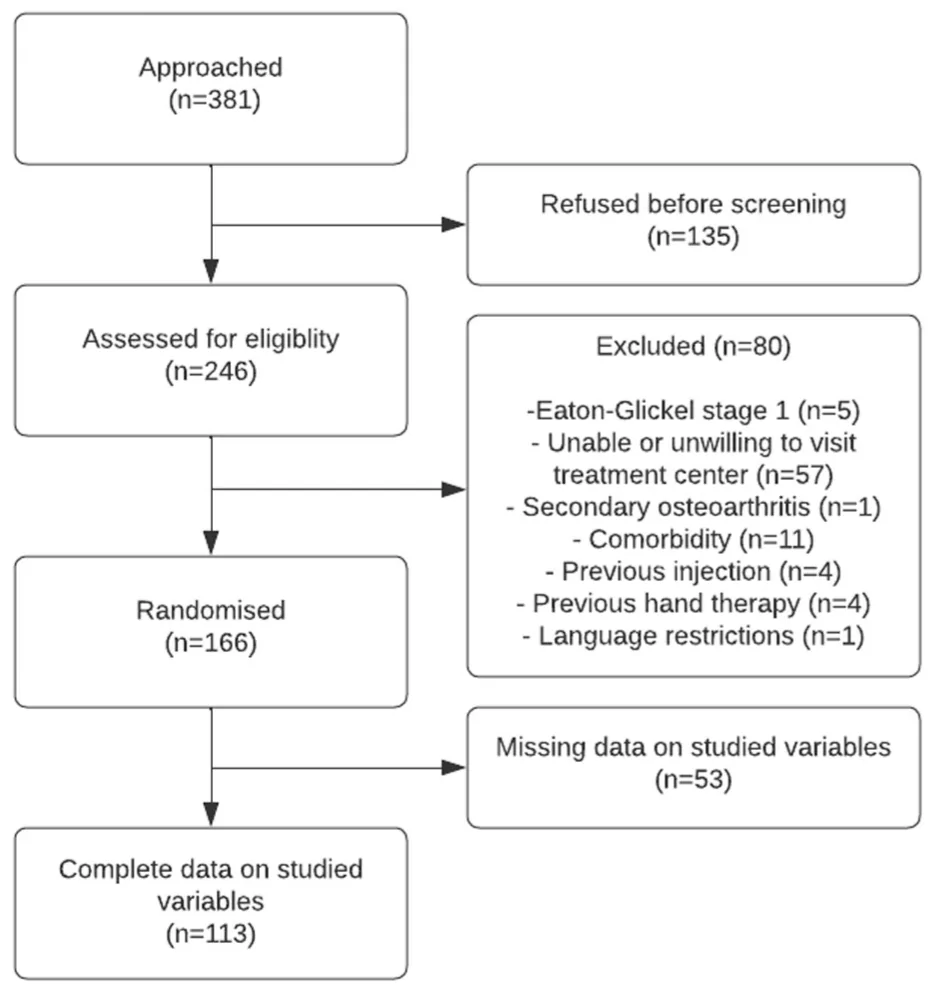
Flowchart of the study.

**Table 1.** Demographic characteristics of the study participants (*n* = 113).

Variable	Category	Results
Age, years		64 (8)
Female sex, *n* (%)		69 (61)
Hand affected, *n* (%)	Left	46 (41)
	Right	40 (35)
	Both	27 (24)
Duration of symptoms in months		42 (81)
Eaton–Glickel stage, *n* (%)	Stage 2	41 (36)
	Stage 3	46 (41)
	Stage 4	6 (5)
	NA	20 (18)
Dominant hand, *n* (%)	Ambidextrous	4 (4)
	Left	16 (14)
	Right	93 (82)
Type of work, *n* (%)	Unemployed (allowance, retired)	43 (38)
	Unemployed (volunteer)	6 (5)
	Light physical labour	36 (32)
	Moderate physical labour	21 (19)
	Heavy physical labour	7 (6)
Educational level, *n* (%)	Primary education	3 (3)
	Lower secondary vocational education	9 (8)
	General secondary education	2 (2)
	Higher secondary education	17 (15)
	Intermediate vocational education	36 (32)
	Bachelor’s degree	28 (25)
	Master’s degree	14 (12)
	Doctorate	4 (4)
Pain at 3 months (MHQ pain subscale; range 0–100)		65 (19)
Pain at baseline (MHQ pain subscale; range 0–100)		52 (18)
Outcome expectations baseline score (CEQ; range 3–37)		18 (5)
Perceived attention score at 3 months (CARE; range 0–50)		4 (1)
Treatment frequency (number of visits in 3 months)		7 (4)
Patient experience with care score at 3 months (PREM; range 0–10)		8 (1)
Depression severity score at baseline (PHQ-9; range 0–27)		2 (3)
Generalized anxiety score at baseline (GAD-7; range 0–21)		2 (3)
Illness perception score at baseline (B-IPQ; range 0–80)		32 (12)
Pain catastrophizing score at baseline (PCS; range 0–52)		9 (8)
Change in grip strength at 3 months (kg)		1 (5)
Change in key pinch at 3 months (kg)		0.5 (1)
Change in tip pinch at 3 months (kg)		0.4 (1)
Therapy adherence score at 6 weeks (TAAT; range 0–5)		3 (1)

Numbers are mean (SD) unless denoted as *n* (%). MHQ: Michigan Hand outcome Questionnaire, adjusted so that higher scores indicate better outcome; CEQ: Credibility and Expectancy Score; CARE: Consultation and Relational Empathy measure; PREM: Patient-Reported Experience Measure; PHQ-9: Patient Health Questionnaire-9; GAD-7: Generalized Anxiety Disorder-7; B-IPQ: Brief Illness Perception Questionnaire; PCS: Pain Catastrophizing Scale; TAAT: Therapy Adherence Assessment Tool.

The non-responder analysis indicated that two out of nine variables differed between responders and non-responders: handedness and educational level (Appendix II, available online). However, the non-significant results of Little’s test (*p* = 0.41) suggest that the data were missing completely at random. Guided by this, we performed a complete case analysis.

### Associations with pain

Three out of 13 variables were significantly associated with a better (higher) MHQ pain score at 3 months: better MHQ pain score at baseline (beta coefficient 0.55 (95% CI: 0.37 to 0.73); *p* < 0.001), higher baseline outcome expectations measured with the CEQ (0.88 (95% CI: 0.18 to 1.58); *p* = 0.014), and improvement in grip strength after 3 months (0.66 (95% CI: 0.06 to 1.26); *p* = 0.032). The final model explained 55% of the variance in MHQ pain scores (*R*² = 0.554), with an adjusted *R*² of 0.496, indicating a moderate fit.

Based on the standardized beta coefficient, the MHQ pain baseline score had the strongest independent association (0.54 (95% CI: 0.36 to 0.71); *p* < 0.001) with pain at 3 months (Figure 2). Univariable associations are presented in Appendix III (available online).

**Figure 2.**
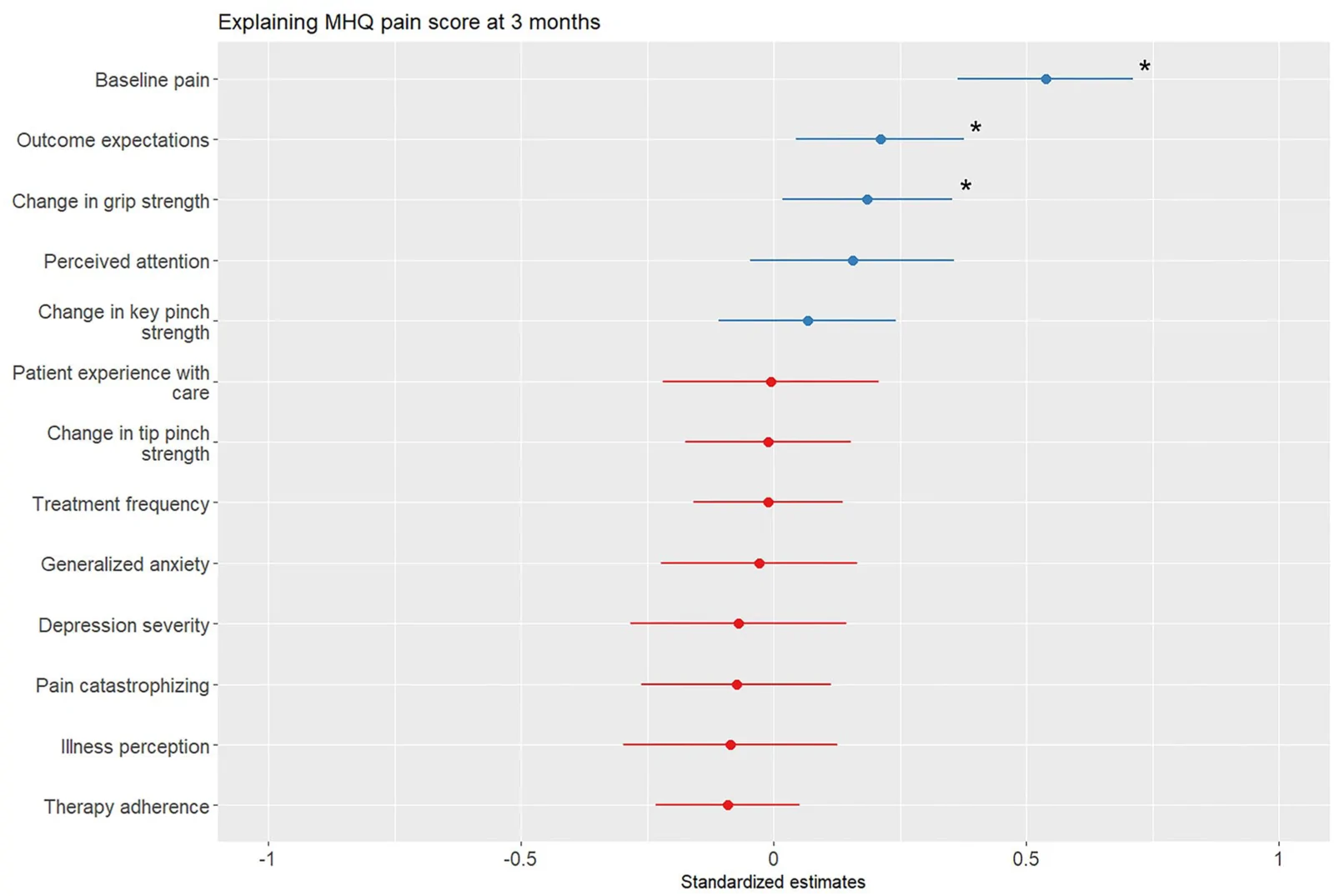
Standardized beta coefficients for each variable showing the association with the Michigan Hand outcome Questionnaire (MHQ) pain subscale at 3 months, and the 95% confidence interval. Variables marked with an asterisk (*) indicate a statistically significant association (*p* < 0.05). Blue lines indicate positive associations and red lines indicate negative associations. Among the 13 predictors, baseline pain score, higher baseline outcome expectations and improvement in grip strength at 3 months showed significant associations with pain at 3 months. The baseline pain score had the strongest independent association with pain at 3 months.

## Discussion

We found that baseline pain severity, higher outcome expectations and improvement in grip strength are associated with pain at 3 months after the use of orthoses with or without exercise therapy for TMJ OA. These findings confirm and reinforce existing evidence on the importance of both initial symptom severity and patient expectations in non-surgical treatment outcomes. The explained variance in our model was higher than that reported in earlier studies, suggesting that the included variables contribute to understanding short-term pain outcomes. This may be because, unlike previous studies, we included not only baseline variables, but also additional factors measured at 6 weeks and 3 months, providing a more comprehensive view of what is associated with pain. Other variables, including psychological factors, adherence to therapy, perceived attention, patient experience with care, treatment frequency and changes in pinch strength were not independently associated with pain improvement in our model.

Our results show that better baseline pain scores (i.e. less severe pain) are strongly associated with better outcomes at 3 months, consistent with previous studies (Tsehaie et al., 2018; van der Oest et al., 2022; Wouters et al., 2019). This finding suggests that patients experiencing considerable pain at baseline are more likely to have residual pain despite treatment, and this is in agreement with studies indicating that baseline symptom severity can shape long-term outcomes in hand osteoarthritis (Bijsterbosch et al., 2011; van der Meulen et al., 2023).

Baseline outcome expectations, as measured by the CEQ, were also significantly associated with pain outcomes at 3 months. This finding supports growing evidence that personal factors, such as treatment expectations, influence treatment success (van der Oest et al., 2022). This success can be reflected not only in pain reduction but also in outcomes such as patient satisfaction (De Ridder et al., 2022; Hoogendam et al., 2021). Therefore, enhancing patient expectations through clear communication and education could be effective in improving treatment outcomes (Loos et al., 2025).

Improvement in grip strength was associated with better pain outcomes, consistent with previous research demonstrating links between joint stability, hand function and pain relief (Thakker et al., 2025; Wouters et al., 2019). Although this may reflect mechanisms, such as reduced joint strain from improved stability, it may also operate through indirect pathways, such as increasing patients’ confidence in using their hands. Nevertheless, this finding supports the potential relevance of incorporating strength-focused interventions in hand therapy to achieve optimal pain management outcomes. However, changes in pinch strength were not associated with pain outcomes. This may be because the variability was too small to detect a meaningful association with pain.

Several psychological factors, including anxiety, depression, illness perception and pain catastrophizing, and also perceived attention and patient experience with care, were significantly associated with pain at 3 months in univariable analyses. However, none remained independently associated in the multivariable model. Although psychological factors are often linked to changes in pain perception (van der Oest et al., 2022), their lack of independent significance here may reflect overlapping variance with stronger predictors.

Furthermore, the low prevalence of psychological distress in this cohort probably limited the statistical power to detect independent associations. The mean baseline scores for the PHQ-9, GAD-7 and PCS were substantially below clinical cut-offs, suggesting that most participants did not experience clinically significant distress. Similarly, the high overall scores for perceived attention and patient experience suggest the possibility of ceiling effects. Such restricted variability may have limited the statistical power to detect independent associations in the multivariable analysis, even when they were evident in univariable testing.

These findings may also indicate that psychological factors influence the degree of change in pain over time rather than determining absolute residual pain at a specific time point. Alternatively, the lack of associations may reflect a stronger relationship between psychological factors and baseline pain rather than with changes over time. Future research could explore this distinction to better understand the mechanisms underlying psychological influences on pain.

Adherence to therapy, measured with the TAAT, was expected to be associated with pain at 3 months, but showed no association in either univariable or multivariable analyses. This unexpected finding may be because of low variability, as most patients reported high adherence to therapy.

This study has several limitations. One limitation is missing data, although this is inherent to research using patient-reported outcome measurements. Our analysis was limited to patients with complete data on all variables of interest. This may have introduced selection bias, since participants with missing data may have differed from those included in the analysis. A non-responder analysis revealed significant differences in two demographic variables between responders and non-responders: dominant hand and educational level. Given that Little’s test suggested that the data were missing completely at random and the overall patterns, we do not believe they affected the outcome of our study.

Our analysis combined patients who received only orthoses with those who also underwent exercise therapy, which may have introduced outcome variability. Although the use of orthoses was standardized, therapy sessions could vary in intensity and delivery. This variability could have masked differences in the outcome of these interventions but it may not be a significant issue as it reflects real-world clinical practice. Moreover, the primary study comparing the two groups found no significant difference in pain (Wouters et al., 2026).

Our study focused on short-term outcomes at 3 months but longer follow-up is necessary to determine whether the identified factors have a sustained impact on pain and function over time.

## Supplemental Material

sj-docx-1-jhs-10.1177_17531934261459874 – Supplemental material for Factors associated with pain after non-surgical treatment for trapeziometacarpal joint osteoarthritis Supplemental material, sj-docx-1-jhs-10.1177_17531934261459874 for Factors associated with pain after non-surgical treatment for trapeziometacarpal joint osteoarthritis by Lisa M J Esteban Lopez, Lisa Hoogendam, Ruud W Selles and Robbert M Wouters in Journal of Hand Surgery (European Volume)

sj-docx-2-jhs-10.1177_17531934261459874 – Supplemental material for Factors associated with pain after non-surgical treatment for trapeziometacarpal joint osteoarthritis Supplemental material, sj-docx-2-jhs-10.1177_17531934261459874 for Factors associated with pain after non-surgical treatment for trapeziometacarpal joint osteoarthritis by Lisa M J Esteban Lopez, Lisa Hoogendam, Ruud W Selles and Robbert M Wouters in Journal of Hand Surgery (European Volume)

sj-docx-3-jhs-10.1177_17531934261459874 – Supplemental material for Factors associated with pain after non-surgical treatment for trapeziometacarpal joint osteoarthritis Supplemental material, sj-docx-3-jhs-10.1177_17531934261459874 for Factors associated with pain after non-surgical treatment for trapeziometacarpal joint osteoarthritis by Lisa M J Esteban Lopez, Lisa Hoogendam, Ruud W Selles and Robbert M Wouters in Journal of Hand Surgery (European Volume)

sj-docx-4-jhs-10.1177_17531934261459874 – Supplemental material for Factors associated with pain after non-surgical treatment for trapeziometacarpal joint osteoarthritis Supplemental material, sj-docx-4-jhs-10.1177_17531934261459874 for Factors associated with pain after non-surgical treatment for trapeziometacarpal joint osteoarthritis by Lisa M J Esteban Lopez, Lisa Hoogendam, Ruud W Selles and Robbert M Wouters in Journal of Hand Surgery (European Volume)
