# Supplementary material for: Factors associated with pain after non-surgical treatment for trapeziometacarpal joint osteoarthritis
Source: J Hand Surg Eur Vol. 2026 Jun 25;51(10):1312–9. doi: 10.1177/17531934261459874 (PMC13672954; doi:10.1177/17531934261459874)
Supplement: Supplementary file 2 [file sj-docx-2-jhs-10.1177_17531934261459874.docx]

**APPENDIX II.** Non-responder analysis was conducted to assess whether patients with missing values differed systematically from those without, by comparing demographics and the primary outcome between patients with data for the studied variables (responders) and those without such data (non-responders).

| **Variable** | **Responders N=113** | **Nonresponders N=53** | **p-value** | **SMD** |
| --- | --- | --- | --- | --- |
| Age, mean (SD) | 64 (8) | 63 (9) | 0.588 | 0.088 |
| Female sex, n (%) | 69 (61) | 37 (70) | 0.357 | 0.185 |
| Hand affected, n (%)  Left  Right  Both | 46 (41) 40 (35) 27 (24) | 18 (34) 18 (34) 17 (32) | 0.507 | 0.192 |
| Duration of symptoms in  months, mean (SD) | 42 (81) | 40 (82) | 0.919 | 0.018 |
| Eaton-Glickel stage, n (%)  Stage 2  Stage 3  Stage 4  NA | 41 (36) 46 (41) 6 (5) 20 (18) | 25 (47) 17 (32) 1 (2) 10 (19) | 0.421 | 0.290 |
| Dominant hand, n(%)  Ambidextrous  Left  Right  NA | 4 (4) 16 (14) 93 (82) 0 (0) | 2 (4) 9 (17) 34 (64) 8 (15) | **<0.001 *** | **0.621** |
| Type of work, n (%)  Unemployed (allowance, retired)  Unemployed (volunteer)  Light physical labor  Moderate physical labor  Heavy physical labor  NA | 43 (38) 6 (5) 36 (32) 21 (19) 7 (6) 0 (0) | 24 (45)  3 (6) 11 (21) 9 (17) 5 (9) 1 (2) | 0.447 | 0.340 |
| Educational level n (%)  Primary education  Lower secondary vocational education  General secondary education  Higher secondary education  Intermediate vocational education  Bachelor’s degree  Master’s degree  Doctoral degree  NA | 3 (3)  9 (8)  2 (2)  17 (15)  36 (32)  28 (25)  14 (12)  4 (3)  0 (0.0) | 1 (2)  7 (13)  1 (2)  5 (9)  14 (26)  12 (23)  5 (9)  0 (0)  8 (15) | **0.005*** | **0.762** |
| MHQ pain subscale 3 months, mean (SD) | 65 (19) | 57 (24) | 0.083 | 0.358 |

MHQ = Michigan Hand outcome Questionnaire, adjusted so that higher scores indicate better outcome**. ** indicate a significant p-value of <0.05*** *SMD=Standardized Mean Difference*
