# Supplementary material for: Factors associated with pain after non-surgical treatment for trapeziometacarpal joint osteoarthritis
Source: J Hand Surg Eur Vol. 2026 Jun 25;51(10):1312–9. doi: 10.1177/17531934261459874 (PMC13672954; doi:10.1177/17531934261459874)
Supplement: Supplementary file 3 [file sj-docx-3-jhs-10.1177_17531934261459874.docx]

**APPENDIX III.** Univariable associations with MHQ pain subscale at three months

| **Predictor** | **Coefficient** | **Std. Error** | **t-value** | **p-value** |
| --- | --- | --- | --- | --- |
| MHQ pain subscale baseline score | 0.618 | 0.078 | 7.92 | **<0.0001*** |
| CEQ outcome expectations baseline score | 1.064 | 0.384 | 2.77 | **0.0065*** |
| CARE score 3 months | 6.545 | 1.935 | 3.38 | **0.00099*** |
| Number of visits | 0.113 | 0.403 | 0.28 | 0.779 |
| PREM score | 3.844 | 1.193 | 3.22 | **0.00167*** |
| PHQ-9 baseline score | -2.053 | 0.555 | -3.70 | **0.00034*** |
| GAD-7 baseline score | -1.422 | 0.552 | -2.58 | **0.0113*** |
| B-IPQ baseline score | -0.876 | 0.130 | -6.72 | **<0.0001*** |
| PCS baseline score | -1.034 | 0.195 | -5.31 | **<0.0001*** |
| Change in grip strength | 0.410 | 0.335 | 1.22 | 0.223 |
| Change in key pinch strength | 0.370 | 1.299 | 0.28 | 0.776 |
| Change in tip pinch strength | 0.687 | 1.499 | 0.46 | 0.648 |
| TAAT score 6 weeks | -0.020 | 2.545 | -0.01 | 0.994 |

MHQ = Michigan Hand outcome Questionnaire, adjusted so that higher scores indicate better outcome, CEQ = Credibility and Expectancy Score, CARE = Consultation and Relational Empathy measure, PREM= Patient-Reported Experience Measure, PHQ-9 = Patient Health Questionnaire-9, GAD-7 = Generalized Anxiety Disorder-7, B-IPQ = Brief Illness Perception Questionnaire, PCS = Pain Catastrophizing Scale, TAAT = Therapy Adherence Assessment Tool.	**** indicate a significant p-value of <0.05***
