# Supplementary material for: Factors associated with pain after non-surgical treatment for trapeziometacarpal joint osteoarthritis
Source: J Hand Surg Eur Vol. 2026 Jun 25;51(10):1312–9. doi: 10.1177/17531934261459874 (PMC13672954; doi:10.1177/17531934261459874)
Supplement: Supplementary file 4 [file sj-docx-4-jhs-10.1177_17531934261459874.docx]

**SUPPLEMENTARY MATERIAL**

**Appendix 1, 2,3**

**Table 1.** Demographic characteristics of the study participants (*n*=113).

| Variable |  |  |
| --- | --- | --- |
| Age, years |  | 64 (8) |
| Female sex, *n* (%) |  | 69 (61) |
| Hand affected, *n* (%) | Left Right Both | 46 (41) 40 (35) 27 (24) |
| Duration of symptoms in months |  | 42 (81) |
| Eaton-Glickel stage, *n* (%) | Stage 2 Stage 3 Stage 4 NA | 41 (36) 46 (41) 6 (5) 20 (18) |
| Dominant hand, *n* (%) | Ambidextrous Left Right | 4 (4) 16 (14) 93 (82) |
| Type of work, *n* (%) | Unemployed (allowance, retired) Unemployed (volunteer) Light physical labour Moderate physical labour Heavy physical labour | 43 (38) 6 (5) 36 (32) 21 (19) 7 (6) |
| Educational level, *n* (%) | Primary education  Lower secondary vocational education  General secondary education Higher secondary education Intermediate vocational education Bachelor’s degree Master’s degree Doctorate | 3 (3) 9 (8)  2 (2) 17 (15) 36 (32) 28 (25) 14 (12) 4 (4) |
| Pain at 3 months (MHQ pain subscale; range 0-100) |  | 65 (19) |
| Pain at baseline (MHQ pain subscale; range 0-100) |  | 52 (18) |
| Outcome expectations baseline score (CEQ; range 3-37) |  | 18 (5) |
| Perceived attention score at 3 months (CARE; range 0-50) |  | 4 (1) |
| Treatment frequency (number of visits in 3 months) |  | 7 (4) |
| Patient experience with care score at 3 months (PREM; range 0-10) |  | 8 (1) |
| Depression severity score at baseline (PHQ-9; range 0-27) |  | 2 (3) |
| Generalized anxiety score at baseline (GAD-7; range 0-21) |  | 2 (3) |
| Illness perception score at baseline (B-IPQ; range 0-80) |  | 32 (12) |
| Pain catastrophizing score at baseline (PCS; range 0-52) |  | 9 (8) |
| Change in grip strength at 3 months (kg) |  | 1 (5) |
| Change in key pinch at 3 months (kg) |  | 0.5 (1) |
| Change in tip pinch at 3 months (kg) |  | 0.4 (1) |
| Therapy Adherence score at 6 weeks (TAAT; range 0-5) |  | 3 (1) |

Numbers are mean (SD) unless denoted as *n* (%). MHQ: Michigan Hand outcome Questionnaire, adjusted so that higher scores indicate better outcome; CEQ: Credibility and Expectancy Score; CARE: Consultation and Relational Empathy measure; PREM: Patient-Reported Experience Measure; PHQ-9: Patient Health Questionnaire-9; GAD-7: Generalized Anxiety Disorder-7; B-IPQ: Brief Illness Perception Questionnaire; PCS: Pain Catastrophizing Scale; TAAT: Therapy Adherence Assessment Tool.

# **APPENDIX I – TREATMENT PROTOCOL**

# THETA Study Treatment Protocol

This guideline describes the treatment of patients participating in the THETA study who will follow the treatment pathway with you. It is crucial in this study that participating patients from both the intervention group and the control group undergo a standardized treatment program, regardless of which participating location they attend.
In this study, there are two treatment groups: a control group and an intervention group. Participants are assigned to one of the two treatment groups by lottery. Patients in the control group are treated with a CMC-1 immobilizing orthosis with MCP-1 support (also known as a butterfly orthosis). In this group, the orthosis is fitted, and an orthosis check will take place, but no further interventions are applied. The intervention group receives the same orthosis and will also be offered exercise therapy for 12 weeks.

This guideline describes the implementation of the hand therapeutic treatment. **The treatment within the THETA study is standardized, which means that this treatment guideline cannot be deviated from.** In the intervention group, homework exercises are also prescribed; these exercises are also standardized and the same for each patient. Additionally, there is reference material concerning joint protection principles that you can use during the treatment.

If you have any questions about the implementation of the treatment, please contact us by email/phone using the details below.

*** MASKED ***

*** MASKED ***

**Treatment – Intervention Group: Orthosis + Exercise Therapy**

Treatment duration:	±12 weeks
Treatment frequency:	±1x per week
Number of sessions:	max. 12 sessions of physical therapy

+ 1 session for orthosis fitting and 1 session for checkup and

adjustment if necessary

**Orthosis**
A handmade butterfly orthosis is fitted (Figure 1).
Points of attention for the orthosis:

- Palmar and radial abduction position of the CMC-1,
  no flexion/adduction
- IP-1 is able to move freely
- No pressure points

*Figure 1: Handmade butterfly orthosis*

After one week, an orthosis check is performed to resolve any pinching or pressure points. At this appointment, the wearing policy is repeated. No further occupational therapy takes place.

Orthosis wearing policy:

- The patient is advised to wear the orthosis as much as possible (24/7) in the first two weeks
- From 2 weeks, the orthosis can be reduced during light activities
- From 6 weeks, the orthosis only needs to be worn during heavy activities if necessary, and its use is reduced

**Exercise Therapy**

Main goal: improving the active stability of the CMC1 joint in a correct pinch position. The aim is always to achieve and/or restore a good stable thumb arch. This thumb arch includes a CMC1 joint in radial and palmar abduction combined with MCP-1 flexion and very light IP-1 flexion (Figure 2). One of the characteristics of the correct thumb arch is that the first metacarpal is always in a ‘higher’ position than the secondmetacarpal.

Exercise Therapy Build-Up - Phase 1 (week 1 to 6):
*Objectives:*

*Figure 2: The correct thumb position*

- Pain reduction
- Learning the correct thumb position
- Improving coordination

*Therapy content in this phase, using the following build-up:*
1). Explanation and goal of correct thumb position
2). Start with coordinated/selective tensioning of the a) EPB, b) APB/APL/OP, and c) FPB (exercises 1-3). Expand coordination with more functional exercises, such as picking up and holding various objects while maintaining the thumb position

4). Static exercises with isometric resistance (manually or using an rubber band)

During each session, the home exercises are repeated and corrected where necessary.

*Further points of attention in this phase:*

- The above build-up from steps 1-4 is essential; only move to the next step if the previous step has been achieved
- All exercises should be largely pain-free to avoid new inflammatory reactions. Fatigue is allowed.
- Discuss and apply joint protection principles as described in Attachment 1
- Avoid activity of the Adductor Pollicis, e.g., during resistance exercises
- Avoid activity of the Extensor Pollicis Longus, e.g., during exercise 1
- If there is limited mobility in CMC1 and/or the first web space, it is possible to perform mild mobilizations in radial or palmar abduction. Note: these mobilizations should not cause extra pain or inflammatory reactions.

## Discuss the use of the orthosis as described above

## Exercise Therapy Build-Up - Phase 2 (week 7 to 12):

## *Objectives*

- Expanding active thumb stability
- Improving strength
- Maintaining active stability during functional use of the thumb
- Reducing orthosis use

*Therapy content in this phase, using the following build-up (after completing Phase 1):*

1. Practicing functional grips while maintaining the correct thumb arch with little force
2. Practice active stability and strength in a closed chain with the correct thumb arch isometrically
3. Practice active stability and strength in an open chain with the correct thumb arch isometrically
4. Practice active stability and strength in an open chain with the correct thumb arch dynamically (e.g., with clothespins)

During each session, the home exercises are repeated and corrected where necessary.

*Further points of attention in this phase:*

- The above build-up from step 1-4 is essential; only move to the next step if the previous step has been achieved
- All exercises should be largely pain-free to avoid new inflammatory reactions. Fatigue is allowed.
- Discuss and apply joint protection principles as described in Appendix 1
- Avoid activity of the Adductor Pollicis, e.g., during resistance exercises
- Avoid activity of the Extensor Pollicis Longus, e.g., during exercise 1
- Discuss the use of the orthosis as described above. In this phase, this means that the orthosis is gradually reduced and only used during heavy activities.

## Homework Exercises

*Week 1*

- Exercise 1 + 2	4-5x per day 10-15 reps

*Week 2-4*

- Exercise 1 to 3	4-5x per day 10-15 reps

*Week 5-6*

- Exercise 1 to 3	4-5x per day 10-15 reps
- Practicing correct thumb arch
  If exercises 1 to 3 go smoothly:
- Exercise 4	2-3x per day 2x10 reps (no adduction force)

*Week 7-8*

- Exercise 1 to 3	4-5x per day 10-15 reps
- Exercise 4	2-3x per day 2x10 reps (no adduction force)
- Exercise 5	2-3x per day 2x10 reps (no adduction force)

*Week 9-10*

- Exercise 1 to 3	4-5x per day 10-15 reps
- Exercise 4	2-3x per day 3x10 reps (no adduction force)
- Exercise 5	2-3x per day 3x10 reps (no adduction force)

*Week 11-12*

- Exercise 1 to 3	4-5x per day 10-15 reps
- Exercise 4	2-3x per day 3x10 reps (no adduction force)
- Exercise 5	2-3x per day 3x10 reps (no adduction force)
- Exercise 6	2-3x per day 2x10 reps (no adduction force)
- Exercise 7	2-3x per day 2x10 reps (no adduction force)

## Considerations:

- Follow the build-up as mentioned above
- If there is limited MCP-1 flexion (≤30°), exercise 3 may be difficult to perform. This may be a reason to omit this exercise if it cannot be done.
- If the exercises go well, it may be possible to progress through this schedule more quickly. However, this is only allowed if all preceding exercises are performed well.
- The exercise sheets on the pages below can be given to the patient; make notes on them as needed and write down the exercise frequency.
- For exercises with rubber band/dynabands: start with the lightest and increase the intensity depending on progress.

**Thumb exercises – phase 1**

**1 – Spread thumb horizontally / palmar abduction (APB / APL / OP)**

A - Starting position:	B - End position:

**2 – Lift thumb (EPB)**
A - Starting position:	B - End position

**3 – Flexing thumb middle joint (FPB)**

A - Starting position:	B - End position

**Thumb exercises – phase 2**

**4 – Pull rubber band with correct thumb arch (thumb supported by index finger):**

**5 – Push thumb while maintaining correct thumb arch (thumb supported by index finger):**

**6 – Pull rubber band with stable thumb (thumb not supported by index finger):**

**7 – Push thumb while thumb remains stable (thumb not supported by index finger)**

**Treatment – Intervention Group: Orthosis-only**
Treatment duration:	±2 weeks
Number of sessions:	1 session for orthosis fitting and 1 session for checkup and

adjustment if necessary

**Orthosis**
A handmade butterfly orthosis is fitted (Figure 1).
Points of attention for the orthosis:

- Palmar and radial abduction position of the CMC-1,
  no flexion/adduction
- IP-1 is able to move freely
- No pressure points

*Figure 1: Handmade butterfly orthosis*

After one week, an orthosis check is performed to resolve any pinching or pressure points. At this appointment, the wearing policy is repeated. No further occupational therapy takes place.

Orthosis wearing policy:

- The patient is advised to wear the orthosis as much as possible (24/7) in the first two weeks
- From 2 weeks, the orthosis can be reduced during light activities
- From 6 weeks, the orthosis only needs to be worn during heavy activities if necessary, and its use is reduced

**NO EXERCISE THERAPY / PHYSICAL THERAPY TAKES PLACE**

**Attachment 1 - Ergonomics and joint protection principles for hand OA**

Ergonomics and joint protection principles can be incorporated into the treatment for individuals with thumb base osteoarthritis. To support you in this, here are some tips. For osteoarthritis, it is important to keep moving sufficiently while avoiding overloading (rest rusts principle).

Joint Protection Principles

Goal: Reducing pain, inflammation, and stress on the joints.

- Recognize and respect pain. Pain is a signal that warns that something is wrong in the body, and it should be responded to appropriately. The pain score (NPRS; 0 no pain—10 extreme pain) should not exceed 4. If it does, it is advisable to perform the activity differently or to stop/take a break.
- Ensure a balance between rest and activity.
- Distribute weight/force over multiple joints. Use both hands if necessary.
- Use large joints instead of small joints. Pick up heavy objects with a flat hand rather than by the handle. Be cautious with hot pans.
- Avoid extreme joint positions. Make a proper thumb arch. Avoid pinching and gripping around thin objects; consider thickening.
- Reduce the force and effort required in activities. Replace actions like wringing with squeezing; for example, squeeze a sponge instead of wringing a cloth for wiping the counter; place the cup down instead of lifting it while pouring; use lightweight kitchen utensils or household appliances.
- Avoid prolonged, static positions.
- Prevent prolonged strain.
- Organize spaces logically so that activities do not require too much effort. If necessary, rearrange kitchen cabinets, place heavy equipment low, and keep frequently used items at working height.
- Use assistive devices; peeler, hook knife, hooks on zippers.
- Wear an orthosis during strenuous activities.

**Attachment 2 – Abbreviations**

APB	-	Abductor Pollicis Brevis

APL	-	Abductor Pollicis Longus

CMC-1	-	First carpometacarpal joint

EPB	-	Extensor Pollicis Brevis

FPB	-	Flexor Pollicis Brevis

IP-1	-	First Interphalangeal joint

MCP-1	-	First Metacarpophalangeal joint

OP	-	Opponens Pollicis

**APPENDIX II.** Non-responder analysis was conducted to assess whether patients with missing values differed systematically from those without, by comparing demographics and the primary outcome between patients with data for the studied variables (responders) and those without such data (non-responders).

| **Variable** | **Responders N=113** | **Nonresponders N=53** | **p-value** | **SMD** |
| --- | --- | --- | --- | --- |
| Age, mean (SD) | 64 (8) | 63 (9) | 0.588 | 0.088 |
| Female sex, n (%) | 69 (61) | 37 (70) | 0.357 | 0.185 |
| Hand affected, n (%)  Left  Right  Both | 46 (41) 40 (35) 27 (24) | 18 (34) 18 (34) 17 (32) | 0.507 | 0.192 |
| Duration of symptoms in  months, mean (SD) | 42 (81) | 40 (82) | 0.919 | 0.018 |
| Eaton-Glickel stage, n (%)  Stage 2  Stage 3  Stage 4  NA | 41 (36) 46 (41) 6 (5) 20 (18) | 25 (47) 17 (32) 1 (2) 10 (19) | 0.421 | 0.290 |
| Dominant hand, n(%)  Ambidextrous  Left  Right  NA | 4 (4) 16 (14) 93 (82) 0 (0) | 2 (4) 9 (17) 34 (64) 8 (15) | **<0.001 *** | **0.621** |
| Type of work, n (%)  Unemployed (allowance, retired)  Unemployed (volunteer)  Light physical labor  Moderate physical labor  Heavy physical labor  NA | 43 (38) 6 (5) 36 (32) 21 (19) 7 (6) 0 (0) | 24 (45)  3 (6) 11 (21) 9 (17) 5 (9) 1 (2) | 0.447 | 0.340 |
| Educational level n (%)  Primary education  Lower secondary vocational education  General secondary education  Higher secondary education  Intermediate vocational education  Bachelor’s degree  Master’s degree  Doctoral degree  NA | 3 (3)  9 (8)  2 (2)  17 (15)  36 (32)  28 (25)  14 (12)  4 (3)  0 (0.0) | 1 (2)  7 (13)  1 (2)  5 (9)  14 (26)  12 (23)  5 (9)  0 (0)  8 (15) | **0.005*** | **0.762** |
| MHQ pain subscale 3 months, mean (SD) | 65 (19) | 57 (24) | 0.083 | 0.358 |

MHQ = Michigan Hand outcome Questionnaire, adjusted so that higher scores indicate better outcome**. ** indicate a significant p-value of <0.05*** *SMD=Standardized Mean Difference*

**APPENDIX III.** Univariable associations with MHQ pain subscale at three months

| **Predictor** | **Coefficient** | **Std. Error** | **t-value** | **p-value** |
| --- | --- | --- | --- | --- |
| MHQ pain subscale baseline score | 0.618 | 0.078 | 7.92 | **<0.0001*** |
| CEQ outcome expectations baseline score | 1.064 | 0.384 | 2.77 | **0.0065*** |
| CARE score 3 months | 6.545 | 1.935 | 3.38 | **0.00099*** |
| Number of visits | 0.113 | 0.403 | 0.28 | 0.779 |
| PREM score | 3.844 | 1.193 | 3.22 | **0.00167*** |
| PHQ-9 baseline score | -2.053 | 0.555 | -3.70 | **0.00034*** |
| GAD-7 baseline score | -1.422 | 0.552 | -2.58 | **0.0113*** |
| B-IPQ baseline score | -0.876 | 0.130 | -6.72 | **<0.0001*** |
| PCS baseline score | -1.034 | 0.195 | -5.31 | **<0.0001*** |
| Change in grip strength | 0.410 | 0.335 | 1.22 | 0.223 |
| Change in key pinch strength | 0.370 | 1.299 | 0.28 | 0.776 |
| Change in tip pinch strength | 0.687 | 1.499 | 0.46 | 0.648 |
| TAAT score 6 weeks | -0.020 | 2.545 | -0.01 | 0.994 |

MHQ = Michigan Hand outcome Questionnaire, adjusted so that higher scores indicate better outcome, CEQ = Credibility and Expectancy Score, CARE = Consultation and Relational Empathy measure, PREM= Patient-Reported Experience Measure, PHQ-9 = Patient Health Questionnaire-9, GAD-7 = Generalized Anxiety Disorder-7, B-IPQ = Brief Illness Perception Questionnaire, PCS = Pain Catastrophizing Scale, TAAT = Therapy Adherence Assessment Tool.	**** indicate a significant p-value of <0.05***
